# Monthly Summary

**Published:** 1877-05

**Authors:** 


### MONTHLY SUMMARY.

---

*Dislocation of the Malar Bone.*—A case of dislocation of the malar bone is under observation at the present time, and is of interest from the very great rarity of its occurence. The dislocation was caused by direct injury, resulting from a fall, and is of a compound character. The wound exists external to the outer canthus of the eye, and it would seem that some projecting body, striking the patient in his descent, was the immediate cause of the injury. On passing the finger into the mouth it is noticed that the lower angle of the bone rests on the outer side of the molar teeth. By making pressure it can be apparently replaced, but so far no attempt at reduction has been considered advisable.—*New York Medical Journal.*

---

*A Secret Pile Remedy.*—Professor Edmund Andrews, of Chicago, says, in the *Journal and Examiner :*

A number of men have been itinerating in Illinois and the adjacent States, treating hemorrhoids by a new method. The secret has been sold to various physicians and other persons, at prices varying from fifty to twelve hundred dollars, and some of the purchasers have left a good practice in the expectation of making a fortune by traveling about and applying the remedy.

Investigation has placed the plan more fully in my possession, and I give it here for the benefit of all concerned.

The first thing is to have a good hypodermic syringe, kept in perfect order, with sharp, delicate pipes. The fluid used is strong carbolic acid dissolved in any bland fixed oil. The proportions are usually as follows :

℞—Crystalized carbolic acid........................ ℨiij.

Pure oil...... ..................................... f℥j.—M

Some of the itinerants use equal parts of the two ingredients, and some of them substitute glycerine instead of oil, and at least one of them has tried a preparation of ergot.

When the piles are internal and not readily brought down, a Sims speculum is employed to uncover them. The operator generally takes only one pile at a time, always selecting the uppermost first, and injects into its interior from four to six drops of the carbolized oil, or rather the oleized carbolic acid. The injection turns the pile white, probably coagulates the blood in its vessels, and results in its shrinking away without the inflammation being severe enough at any one time as a general thing, to prevent the patient from attending to his business. The well-known power of carbolic acid to act as a local anæsthetic, antiplogistic, and antisuppurative, favors the progress.

When the irritation of the first injection has measurably subsided, another pile is attacked in the same way, and as the patient cannot see the syringe, he supposes that he has not been subjected to any " operation," which is a great satisfaction to him

The itinerants often call their plan "painless," but it proves in some persons atrociously distressing. The result is in many other cases, excellent, so that the plan may turn out to be worthy of a permanent place in the treatment of hemorrhoids. —*Medical and Surgical Reporter.*

---

*Local Action of Astringents upon the Vessels.*—The mesenteries of curarized frogs were the field of experiment. The transparent membranes were extended over the ring in the cork table, and the selected vessels accurately measured by means of the ocular micrometer ; then a uniformly large drop measured by the pipette, of the 0.1-50.0 per cent. solution of the astringent was applied to the vessel. The metronome was immediately started, and thus the time between the application and any change in the vessels was accurately determined. The following substances were employed :

1. Nitrate of Silver.—This agent retained its ancient reputation, for contraction of the vessels, the veins as well as the arteries without any previous dilatation, to the half of their original diameter took place in from sixteen to eighty seconds after the application, the time varying according to the strength of the solution.

2. Tannic, Gallic and Pyrogallic acids, instead of producing any constriction of the vessels with retardation of the blood current within them, caused dilation of the arteries, capillaries and veins to double their original size. The same effects were produced after the removal of the cord, destruction of the medulla, and even of the brain, so that they were not of reflex origin.

3. Acetate of Lead acted in the same way upon the vessels, with the exception of the capillaries, as the Nitrate of Silver, though in a less powerful degree. The formation of white coagula in the center of the vessels was also frequent, and the author concludes that these are due to increased adhesiveness of the white corpuscles, thus causing a greater tendency than normal to coagulation.

4. The Sequi-chlorine of Iron, unless in 50.0 per cent. solution, produced no effect upon the vessels, and even in that strength was much less powerful than the Silver and Lead salts.

5. Alum.—The experiments with this substance were negative in results, as it produced sometimes contraction, sometimes dilatation, and at other moments no change whatever could be observed.—*Pharmak Uhtersuch.—N. Y. Medical Journal.*

---

*A Young Mother.*—An esteemed and entirely trustworthy correspondent has furnished us with the following facts touching a case which came under his observation. As an instance of early maternity, the case is one which certainly vies with any case on record.

The girl first menstruated when ten years and six months of age. She became pregnant at eleven years and six months, and was safely delivered of a male child January 19th, 1875. The reputed father of the child was, at the time, a hopeful of fourteen years of age. The child is still alive, but not very strong or bright, although the promising parents are doing as well as could be expected.—*Detroit Med. Journal.*

---

*New Observations Concerning the Therapeutic Action of Carbolized Camphor.*—Dr. Soulez publishes in *La France Medicale*, of January 23, 1877, a series of cases in which he successfully employed the carbolized camphor. This antiseptic, of which he speaks so highly, is obtained by dissolving nine grammes of the crystals of carbolic acid in one gramme of alcohol; to two grammes of this solution is then added twelve grammes of powdered camphor—the syrup-like liquid which results is the carbolized camphor. For dressings the carbolized camphor is reduced (one part to nineteen of olive oil or oil of sweet almonds) and in the mixture are saturated square pieces of lint, which are then applied, one over the other, to the wound; these are covered by a thin sheet of india-rubber, and on this, again, dry lint is bound with a bandager M. Soulez claims for this dressing three great advantages: suppression of pain after the operation, absence of febrile reaction, and, finally, rapid cicatrization.—*Clinic.*

